# Comparison of MAPK specificity across the ETS transcription factor family identifies a high-affinity ERK interaction required for ERG function in prostate cells

**DOI:** 10.1186/s12964-015-0089-7

**Published:** 2015-02-19

**Authors:** Nagarathinam Selvaraj, Vivekananda Kedage, Peter C Hollenhorst

**Affiliations:** Medical Sciences, Indiana University School of Medicine, 1001 E 3rd St, Bloomington, IN 47405 USA

**Keywords:** Mitogen activated protein kinase, ERK, JNK, p38, ETS transcription factors, ERG, Prostate cancer, Cell migration

## Abstract

**Background:**

The RAS/MAPK signaling pathway can regulate gene expression by phosphorylating and altering the function of some, but not all, ETS transcription factors. ETS family transcription factors bind similar DNA sequences and can compete for genomic binding sites. However, MAPK regulation varies across the ETS family. Therefore, changing the ETS factor bound to a cis-regulatory element can alter MAPK regulation of gene expression. To understand RAS/MAPK regulated gene expression programs, comprehensive knowledge of the ETS family members that are MAPK targets and relative MAPK targeting efficiency across the family is needed.

**Results:**

An in vitro kinase assay was used to rank-order 27 human ETS family transcription factors based on phosphorylation by ERK2, JNK1, and p38α. Many novel MAPK targets and specificities were identified within the ETS family, including the identification of the prostate cancer oncoprotein ERG as a specific target of ERK2. ERK2 phosphorylation of ERG S215 required a DEF docking domain and was necessary for ERG to activate transcription of cell migration genes and promote prostate cell migration. The ability of ERK2 to bind ERG with higher affinity than ETS1 provided a potential molecular explanation for why ERG overexpression drives migration of prostate cells with low levels of RAS/ERK signaling, while ETS1 has a similar function only when RAS/ERK signaling is high.

**Conclusions:**

The rank ordering of ETS transcription factors as MAPK targets provides an important resource for understanding ETS proteins as mediators of MAPK signaling. This is emphasized by the difference in rank order of ERG and ETS1, which allows these factors to have distinct roles based on the level of RAS/ERK signaling present in the cell.

**Electronic supplementary material:**

The online version of this article (doi:10.1186/s12964-015-0089-7) contains supplementary material, which is available to authorized users.

## Introduction

Most transcription factors can be grouped into families based on common DNA binding domains [1] leading to similar DNA sequence preferences [2]. Through binding site competition, changes in the level of one family member can alter the relative portion of time each other family member occupies a specific location in the genome. If family members have distinct transactivation functions, this competition will alter the expression of target genes. Furthermore, if distinct signaling pathways regulate the transactivation function of different family members, this switch can change the signaling requirements for target gene expression. Predicting how these changes will alter gene expression requires direct functional comparisons.

There are 28 genes encoding ETS family transcription factors in humans. All ETS factors have a winged helix-turn-helix DNA binding domain that allows monomeric binding to the sequence GGA(A/T) [3]. However, ETS factors vary considerably outside of the DNA binding domain, allowing diverse trans-regulatory roles. ETS family transcription factors are extensively co-expressed, with at least 15 members expressed in any cell type [4]. Genomic mapping of ETS targets in a variety of cell types have identified competition for some target sequences between family members [5,6]. A variety of ETS transcription factors have been identified as mediators of RAS/MAPK signaling [7]. Phosphorylation of ETS proteins by distinct classes of MAPK, including ERK, JNK, and p38, can alter transcriptional functions by various mechanisms including changing DNA affinity [8], increasing co-activator recruitment or activity [9,10], or altering subcellular localization [11,12].

Knowing that an ETS factor is a MAPK target is important for understanding regulatory mechanisms. For example, we have recently demonstrated that competition between ETS proteins for a RAS-response element consisting of neighboring ETS and AP-1 binding sites alters cancer cell migration. The ubiquitously expressed ETS protein ETS1 binds ETS/AP-1 sequences in the enhancers of cell migration genes [13]. ETS1 is phosphorylated by ERK when ERK signaling is high, and this increases the transactivation potential of ETS1 and increases expression of cell migration-promoting genes. However, in about one-half of prostate tumors, the ETS protein ERG is over-expressed due to a chromosomal rearrangement [14]. This results in ERG replacing ETS1 at ETS/AP-1 sequences and activating expression of these genes in cells with high levels of PI3K/AKT signaling, but low levels of ERK signaling [15,16]. Therefore, the replacement of ETS1 with ERG alters the signaling requirement for activation of this gene expression program.

Despite the importance of MAPK signaling in the ETS family, it is unknown how many of the 28 human ETS family members can respond to this signaling pathway. Multiple studies have identified individual ETS proteins as a MAPK targets [7]. However, whether or not these particular ETS factors are strong or weak targets compared to all of the other family members that are present in the same cell remains unknown. Here we present the first comprehensive comparison of MAPK targeting within the ETS family. 27 full-length human ETS proteins were purified, and in vitro kinase assays tested phosphorylation specificity for three MAPKs (ERK2, JNK1, and p38α). We provide a rank-ordered comparison of MAPK specificity across the ETS family, confirm known targets and identify many new targets. One striking new observation is the phosphorylation of ERG by ERK. We found that ERK targets S215 of ERG both in vitro and in vivo by interacting with a neighboring DEF domain. This phosphorylation event was critical for the ability of ERG to increase cell migration of prostate epithelial cells. Furthermore, using biochemical methods we determined the affinity of ERK2 for ERG is greater than that for ETS1. This relatively higher affinity correlates with the ability of ERG but not ETS1 to function in cells with low levels of ERK signaling. This example demonstrates the utility of direct comparisons of kinase targeting within a transcription factor family.

## Results

### Comparing MAPK phosphorylation of all human ETS factors

**Figure 1:**
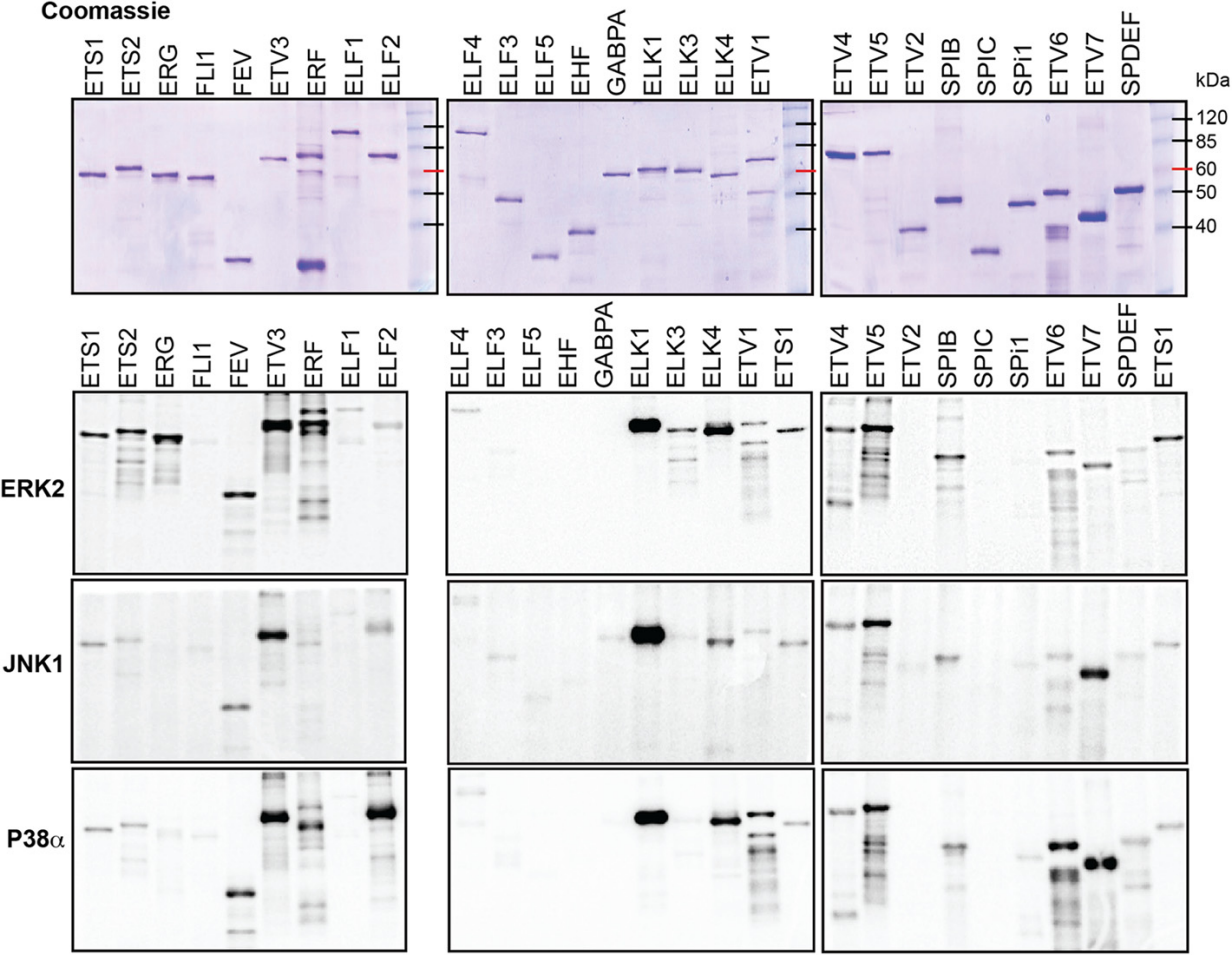
**MAPK specificity across the ETS family.** Coomassie staining of purified ETS proteins (top) or autoradiograph of ^32^P labeled ETS proteins by ERK2, JNK1, or P38α kinase.

To compare ETS family transcription factors, full-length cDNAs of each human ETS gene were expressed in *E. coli* with a 6x Histidine tag on the N-terminus. For ETS genes that express multiple transcripts, we chose versions commonly described in the literature, and biased our selections towards longer versions to include as many potential MAPK target sequences as possible. The transcripts and primer sequences used are in (Additional file 1: Table S1). In sum, 27 ETS proteins were purified from inclusion bodies, solubilized in 5 M urea and then re-folded (Figure 1, top). 25 ETS proteins were soluble in wild-type form after re-folding. Previous studies had reported that the ETS protein ETV6 (TEL) can polymerize through a PNT domain and is insoluble when purified [17,18]. Similarly, the related ETS protein ETV7 (TEL2) is also known to self-associate [19] and, thus, is likely to be insoluble. For this reason, we expressed and purified human ETV6 with an A93D mutation, which in murine ETV6 disrupts polymerization and results in a soluble protein [17]. An analogous mutation (A86D) in human ETV7 was also utilized. Both mutant proteins were soluble and used in place of ETV6 and ETV7 in this study. For all 27 ETS proteins, DNA binding activity in an EMSA confirmed proper folding (Additional file 2: Figure S1).

A 28th ETS family member named *ETV3L* is predicted from genomic sequence, but has not been characterized. Unlike the other 27 ETS family members, we were not able to amplify *ETV3L* cDNA, nor express ETV3L protein from a synthetic construct. For these reasons, it is unclear if *ETV3L* represents an expressed protein-coding gene in humans and we focused on the other 27 ETS family members.

Each ETS transcription factor was used in an in vitro kinase assay with radiolabeled ATP and purified ERK2, JNK1, or p38α kinase (Figure 1). Radiolabel density for the band representing each full-length ETS factor was normalized to coomassie stain density of the same band in the same gel (Additional file 3: Figure S2), and then normalized to the ratio for the ETS1 protein, which was included as a loading control on every gel. This normalized score was then used to rank-order each ETS protein based on the extent of *in vitro* phosphorylation by each MAPK (Tables 1, 2 and 3). We avoid an arbitrary cut-off to report any ETS protein as phosphorylated or not, because such a conclusion would vary based on the activity and local concentration of the kinase *in vivo*. A comparison with previous reports identifying in vitro phosphorylation of ETS proteins by the same MAPK families shows that these findings cluster near the top of our rank order, particularly in the case of the well-studied ERK-ETS interactions (Tables 1, 2 and 3).

**Table 1:** **ERK2 kinase specificity in the ETS family**

**Protein**	**Score**	**Previous report**
ELK1	22.7	Yang et al. [20]
ETV5	9.0	Janknecht et al. [21]
ERF	8.9	Le Gallic et al. [22]
ELK4	8.4	Strahl et al. [23]
ETV3	6.9	Carlson et al. [8]
ERG	2.0	
FEV	1.3	
ELK3	1.2	Ducret et al. [24]
SPIB	1.2	Mao et al. [25]
ETS1	1.0	Yang et al. [26]
ETS2	1.0	Yang et al. [26]
ETV7	0.8	
ETV4	0.8	O’Hagan et al. [27]
ETV1	0.8	Janknecht [28]
ETV6	0.7	Maki et al. [29]
ELF4	0.7	
GABPA	0.2	Flory et al. [30]
ELF2	0.2	
ELF1	0.1	
SPDEF	0.1	
ELF3	0.1	
FLI1	0	
ELF5	0	
ETV2	0	
SPIC	0	
SPI1	0	
EHF	0	

The phosphorylation score is the ratio of radiolabel to total protein normalized to ETS1. The third column references a previous report of in vitro phosphorylation by any member of the ERK MAPK family, if applicable.

**Table 2:** **JNK1 kinase specificity in the ETS family**

**Protein**	**Score**	**Previous report**
ELK1	44.4	Yang et al. [20]
ETV3	19.2	
ETV7	7.0	
ELK4	6.5	
ETV5	6.4	
FEV	2.3	
ERF	2.3	
SPIB	1.7	Mao et al. [25]
ELF2	1.7	
ETV4	1.5	
ETS1	1.0	Paumelle et al. [31]
ETS2	1.0	Fowles et al. [32]
ETV6	0.9	
SPDEF	0.6	
SPI1	0.6	Mao et al. [25]
ELK3	0.5	Ducret et al. [24]
ELF4	0.5	
ETV1	0.4	Janknecht [28]
ELF3	0.3	
GABPA	0.3	Hoffmeyer et al. [33]
FLI1	0.3	
EHF	0.3	
ETV2	0.2	
ELF1	0.2	
ELF5	0.2	
ERG	0.2	
SPIC	0	

Scoring and previous reports for JNK, as in Table 1.

**Table 3:** **p38α kinase specificity in the ETS family**

**Protein**	**Score**	**Previous report**
ETV3	31.9	
ELK1	23.4	Yang et al. [20]
ELF2	13.3	
ETV7	12.0	
ELK4	11.0	Whitmarsh et al. [34]
FEV	7.1	
ETV6	5.2	
ETV5	4.9	
ERF	4.9	Polychronopoulos et al. [35]
ETV1	3.9	Janknecht [28]
SPIB	1.9	
ELF4	1.2	
ETV4	1.1	
ETS2	1.1	
SPDEF	1.0	
ETS1	1.0	Paumelle et al. [31]
SPI1	0.5	
ELK3	0.4	Ducret et al. [24]
ERG	0.4	
FLI1	0.3	
ELF1	0.2	
GABPA	0.2	
ELF3	0.1	
ELF5	0	
ETV2	0	
SPIC	0	
EHF	0	

Scoring and previous reports for p38, as in Table 1.

### Role of MAPK interaction motifs in ETS family specificity

For many of the ETS proteins, ERK2, JNK1 and p38α produced very similar phosphorylation signals (Figure 1), reflected in similarities in the rank order (Tables 1, 2 and 3). MAPKs can bind target proteins by docking to D or DEF domains [36] and then phosphorylating neighboring serine or threonine residues, within consensus PX(TP/SP) motifs, or sometimes within TP or SP motifs that lack a preceding proline. To identify motifs corresponding to phosphorylation by all three MAPKs, in Table 4 we sorted the ETS proteins by ascending mean rank order for all three MAPKs, and listed the number of predicted MAPK interaction domains and potential target residues. This analysis identified a strong correlation between a high level of phosphorylation by all three kinases and the number of PX(TP/SP) motifs in the protein. For example, seven ETS proteins have more than one PX(TP/SP) motif, and these include all of the top five in Table 4. Eleven ETS proteins lack a PX(TP/SP) motif, and these include nine of the bottom 10 in Table 4. Therefore, PX(TP/SP) motifs likely target phosphorylation by all three MAPKs, and a higher number of these motifs leads to more phosphorylation sites and a higher signal in the assay.

**Table 4:** **Presence of MAPK interaction motifs in the ETS family**

**ETS**	**Mean Rank**	**D Domain**	**FXF**	**FXFP**	**PX(S/T)P**	**SP/TP**
ELK1	1.3	2	1	1	3	14
ETV3	2.7	4	3	1	2	12
ELK4	4.3	1	1	1	3	18
ETV5	5.0	3	0	0	3	6
ERF	6.0	2	5	2	7	18
ETV7	6.3	2	1	0	1	7
FEV	6.7	1	1	0	1	2
ELF2	9.7	4	0	0	1	16
SPIB	9.7	0	1	0	1	3
ETV6	11.7	3	1	0	2	16
ETV4	12.0	2	0	0	1	7
ETS1	12.3	3	0	0	0	4
ETS2	12.3	2	0	0	0	5
ETV1	14.0	3	1	0	2	6
ELK3	14.3	4	1	1	1	15
ELF4	14.7	4	0	0	1	8
SPDEF	16.3	2	0	0	1	8
ERG	16.3	2	1	1	0	10
SPI1	19.3	1	1	0	0	3
GABPA	20.3	2	0	0	0	2
ELF3	21.0	0	1	0	0	2
FLI1	21.0	2	0	0	0	4
ELF1	21.0	6	1	0	1	12
ELF5	24.0	0	0	0	0	1
EHF	24.7	2	1	0	0	1
ETV2	25.0	2	1	1	0	3
SPIC	26.0	1	1	0	0	2

ETS proteins are listed by the mean ranking for ERK2, JNK1, and p38α from Tables 1, 2 and 3. The number of indicated motifs is listed. Sequence of D domains is detailed in (Additional file 4: Table S2).

While many ETS proteins were phosphorylated in a similar manner by each MAPK, there were several interesting exceptions. For example, ELF2 and ETV6 were more phosphorylated by p38α than by ERK2 or JNK1 (Figure 1). The D domain is considered an interaction domain for all three MAPK classes. However, p38 kinases have additional acidic residues in their docking regions [37] and the introduction of basic residues into target proteins can promote p38 specificity [38]. Therefore, we searched for basic residues in the D domains of the ETS proteins that might interact with p38α. One part of the D domain, called the LXL motif, consists of two hydrophobic amino acids surrounding a variable residue. The D-Finder algorithm [39] was used to identify 60 D domains in the 27 ETS protein sequences (Additional file 4: Table S2). Of these 60, only two had a basic arginine residue in the central, variable position of the LXL motif (LRL). These two D domains were in ELF2 and ETV6, the same two ETS factors with p38α specificity. Therefore an LRL motif may contribute to p38 specific kinase targeting.

Another group of ETS proteins were more efficiently phosphorylated by ERK2 than by JNK1 or p38α. These included ETS1 and ETS2, which had slight specificity, and ELK3 and ERG, which showed strong specificity for ERK2 (Figure 1). Unlike the D domain, the DEF domain shows preference for ERK compared to JNK or p38 [40,41]. The consensus DEF domain sequence (FXFP) occurs in only seven ETS proteins (Table 4). Among these are four ETS proteins with multiple PX(TP/SP) motifs, where an ERK-specific phosphorylation event would be difficult to detect due to less-specific phosphorylation at other sites. Of the twenty ETS proteins with zero or one PX(TP/SP) motif, only three had an FXFP sequence, and two of these three were ELK3 and ERG, the proteins where we observed strong ERK2 specificity. Therefore, the DEF domain correlates with ERK specificity in the ETS family.

### ERK phosphorylates ERG S215 via a DEF domain

**Figure 2:**
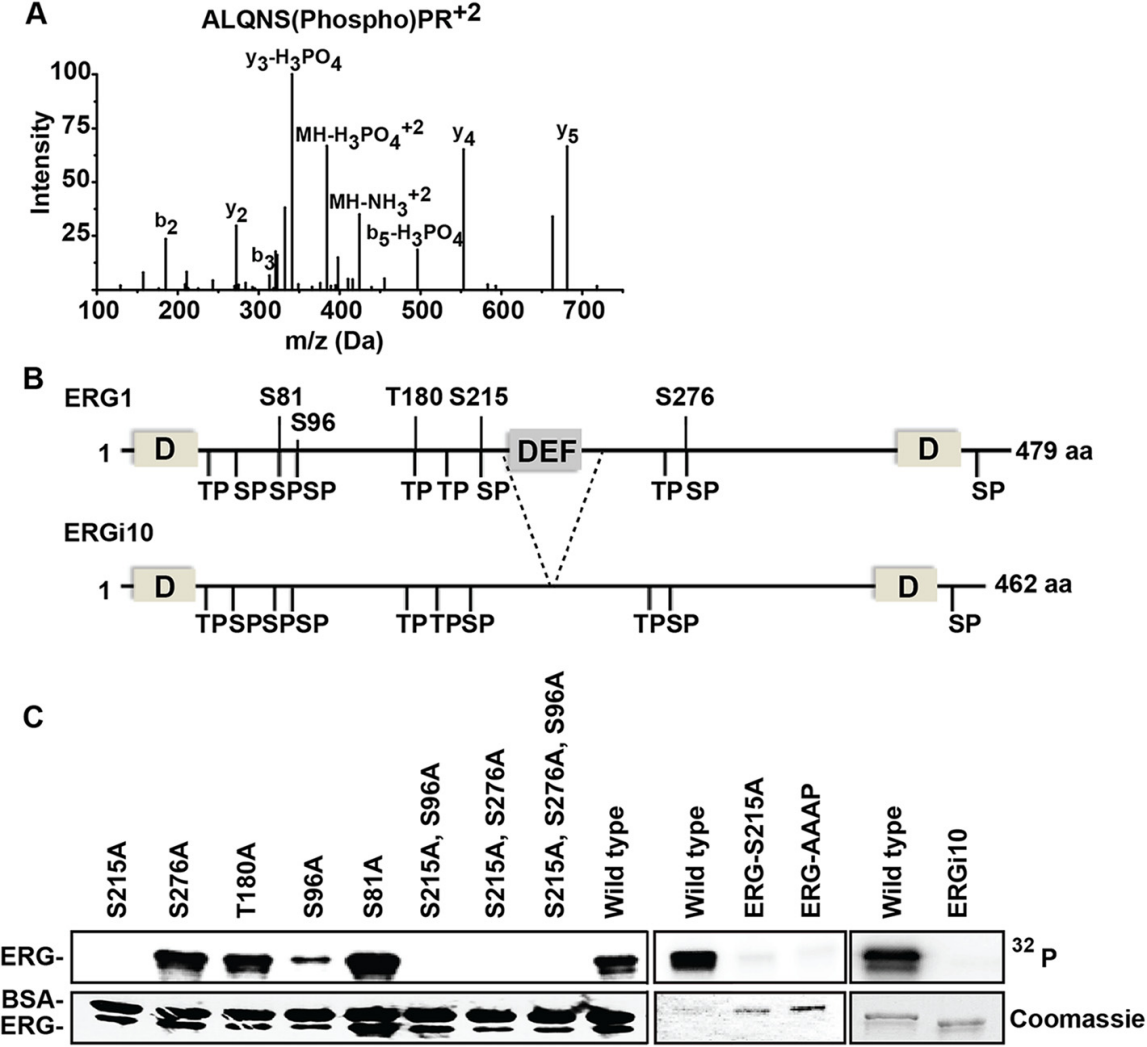
**ERG is phosphorylated at S215 by ERK via an FXFP motif. (A)** Mass spectrum of ERK2 phosphorylated ERG peptide fragment (ALQNS*PR) identifying phosphorylation of S215. **(B)** Schematic representation of ERG and ERGi10 protein isoforms showing possible ERK binding motifs (D and DEF domains, indicated as boxes) and potential phosphorylation sites (TP and SP sequences, indicated with lines). Serine and threonine residues interrogated in **(C)** are annotated. Dashed lines define the region coded by exon 9. **(C)** In vitro kinase assays, as in Figure 1, show ERK2 phosphorylation of the indicated proteins. Note that bovine serum albumin (BSA) was included in the kinase buffer in the left panels, but had no effect on the specificity of the assay (compare to center panel), and was therefore not used in other kinase assays.

The results provided in Figure 1 and Tables 1, 2 and 3 allow comparison of relative phosphorylation efficiency between ETS family members in vitro. To test whether these results can provide important information regarding differences in in vivo ETS functions, we focused on the ability of ERK2 to phosphorylate ERG. ERG is the ETS protein with the highest level of ERK2 phosphorylation that had not been previously reported in the literature (Table 1). To identify the ERK2 target residue, purified ERG was phosphorylated by ERK in vitro, and analyzed by mass spectrometry. Three potential phosphorylation sites were identified (Figure 2A and B). These were S215, S276, and S96. To test the requirement of these residues for in vitro phosphorylation by ERK2, ERG proteins were purified with alanine substitutions at each of these residues, plus two additional nearby TP or SP sequences, T180 and S81. Of these five substitutions, only S215A completely ablated the phosphorylation signal (Figure 2C, left panels), indicating that S215 is the major ERK2 phosphorylation site. The S96A mutation reduced the signal, indicating a potential secondary site that requires prior S215 phosphorylation. Significantly, S215 represents the nearest SP or TP sequence to the DEF domain found in ERG (Figure 2B). To test the role of the DEF domain, ERG was purified with a mutation in this domain (FIFP to AAAP). An in vitro phosphorylation assay showed that both S215 and DEF domain mutations ablate the ability of ERK2 to phosphorylate ERG (Figure 2C, center panels), indicating that S215 is targeted by ERK2 binding to the DEF domain in vitro. In cloning ERG (isoform 1), we also cloned an alternatively spliced version of ERG lacking Exon 9 (isoform 10, ERGi10). ERGi10 lacks 24 amino acids including the DEF domain (Figure 2B), but retains S215. Purified ERGi10 was not phosphorylated, consistent with a requirement for the DEF domain within Exon 9 (Figure 2C, right panels).

**Figure 3:**
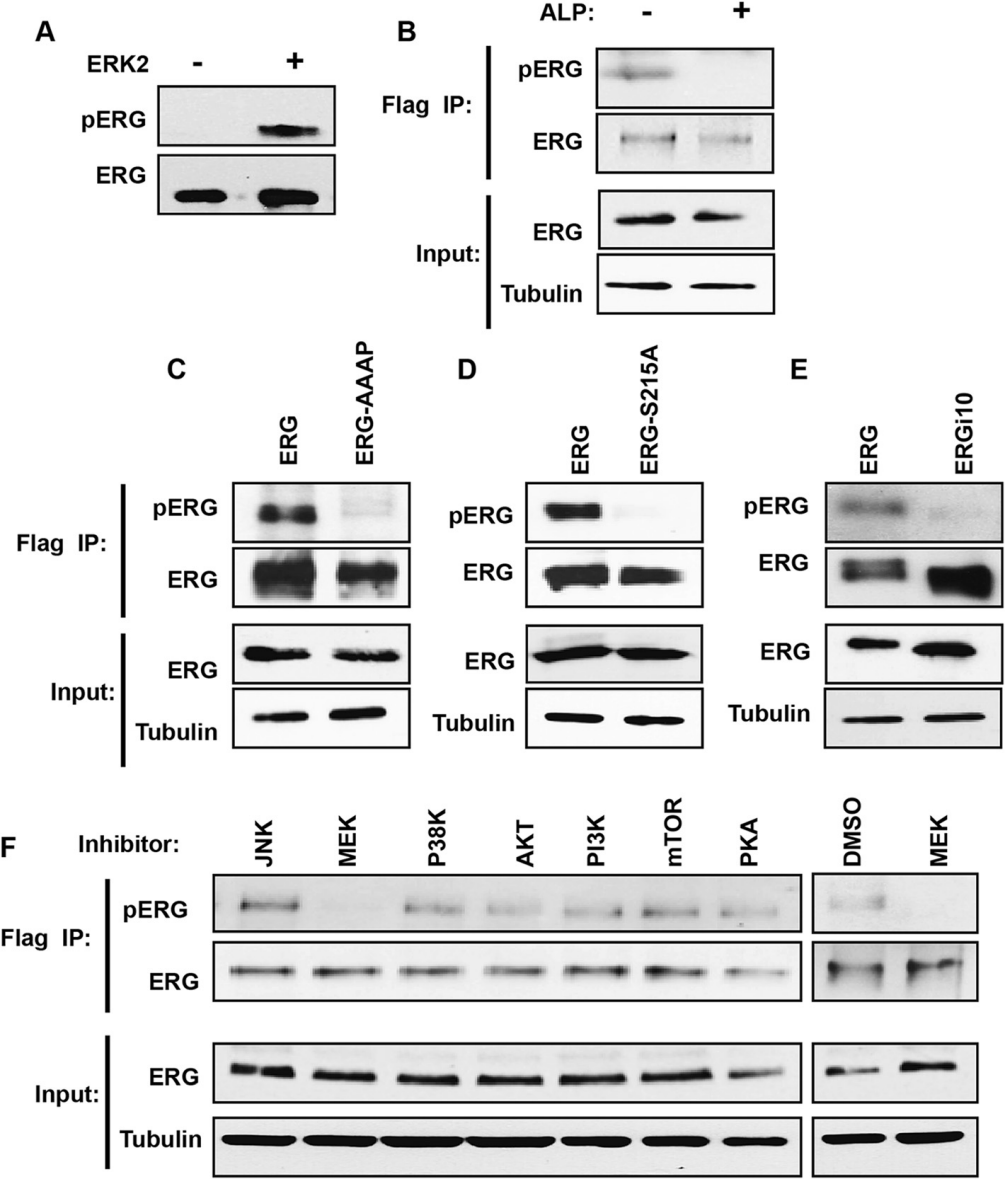
**ERG is phosphorylated at S215 by the RAS/MEK/ERK pathway in prostate epithelial cells. (A)** Immunoblot of purified ERG protein with phospho-S215 pERG antibody after phosphorylation by ERK, or mock treatment. **(B)** Immunoblot with pERG or total ERG antibodies of whole cell extract, or protein immunoprecipitated with Flag antibody from Flag-ERG expressing RWPE1 cells. Immunoprecipitates were treated with alkaline phosphatase as indicated. **(C, D, E)** Immunoblots as in **(B)**, but from RWPE1 cells stably expressing the indicated ERG construct. **(F)** Immunoblots as in **(B)**, but from cells treated with the indicated inhibitors, or DMSO as a negative control.

A previous mass spectrometry analysis of ERG immunoprecipitated from prostate cancer cells with a TMPRSS2/ERG fusion, identified S215 phosphorylation [42], indicating that this modification can occur in vivo. To measure S215 phosphorylation in vivo, a phospho-specific antibody to ERG S215p was raised (pERG antibody). This antibody detected purified ERG protein only after it was phosphorylated by ERK2 (Figure 3A). The RWPE-ERG cell line was then used to identify ERG phosphorylation in vivo. RWPE1 cells are derived from normal prostate epithelia and do not express ERG. Stable expression of 3xFlag tagged ERG via a retroviral vector creates RWPE-ERG cells. ERG was immunoprecipitated from RWPE-ERG cells with the Flag antibody, then immunoblotted with the ERG and pERG antibodies. The pERG antibody detected a band the same size as ERG, and this band disappeared when the extract was treated with alkaline phosphatase, indicating that it detected a phosphorylation site (Figure 3B). RWPE1 cells stably expressing ERG with S215A and AAAP mutations or ERGi10 were then compared to RWPE-ERG cells. Both mutant ERG proteins and ERGi10 were expressed at similar levels to ERG, but were not detected by the pERG antibody (Figure 3C to E). These findings indicate that the pERG antibody is specific for ERG S215 phosphorylation, and the ERG DEF domain is required for S215 phosphorylation in vivo.

To identify the signaling pathway that results in ERG S215 phosphorylation in vivo, RWPE-ERG cells were treated with eight different kinase or signaling pathway inhibitors. The only inhibitor to reduce S215 phosphorylation was the MEK inhibitor (Figure 3F). MEK phosphorylation of ERK is necessary for ERK kinase function, so these results are consistent with ERK being the kinase that phosphorylates ERG S215 in vivo.

### S215 phosphorylation is necessary for ERG to promote prostate cell migration

**Figure 4:**
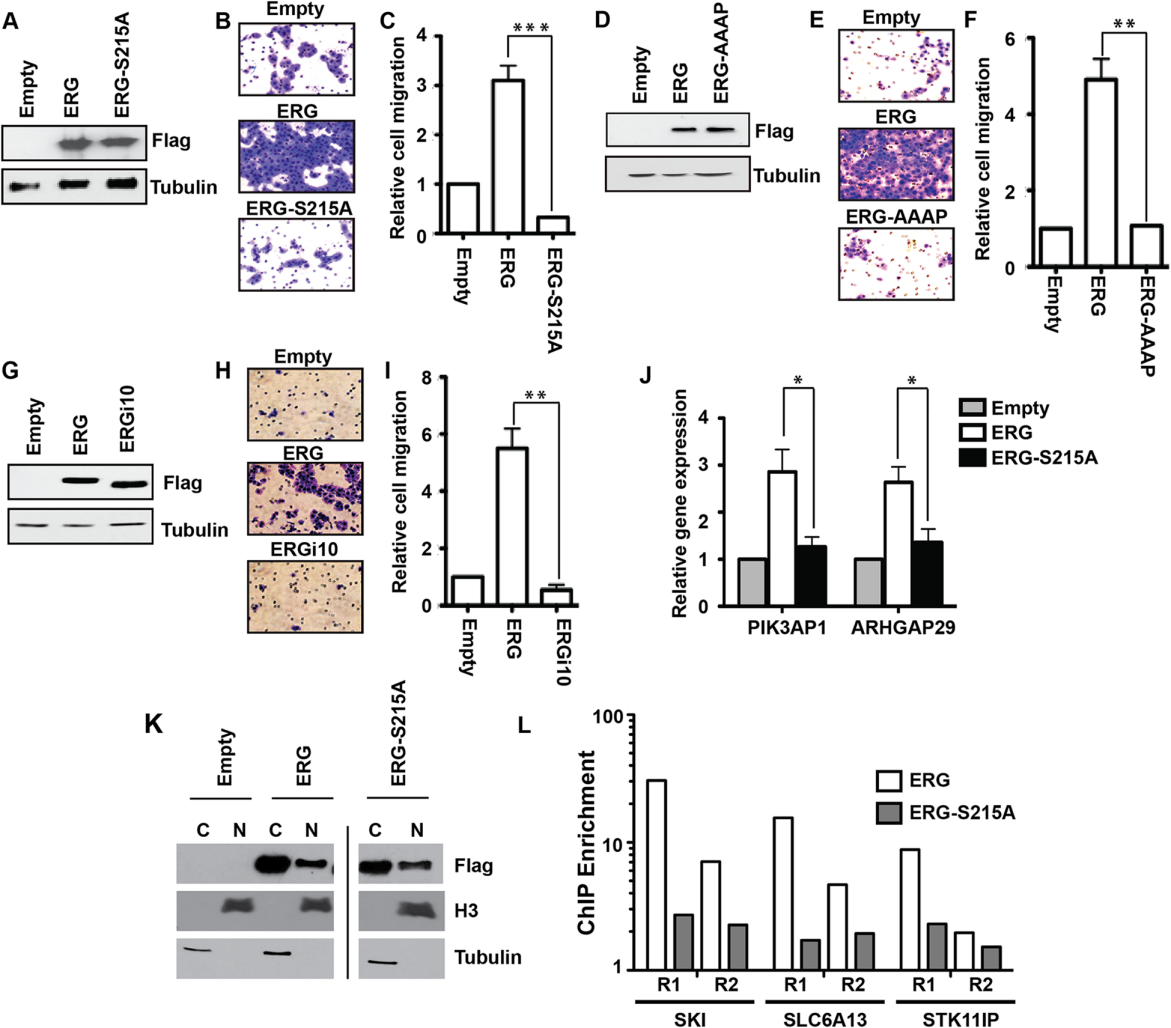
**ERG requires S215 and the FXFP motif to promote prostate cell migration and activate target genes. (A, D, G)** Flag Immunoblots of RWPE1 cells stably expressing the indicated Flag-ERG proteins. **(B, E, H)** Representative images of transwell migration assays in the given cell lines **(C, F, I)** Quantification of transwell assays shows relative number of cells migrated compared to empty vector as the mean and SEM of at least three biological replicates. **(J)** qRT-PCR measured relative mRNA levels of migration related genes in ERG expressing RWPE1 cells compared to those expressing vector only. Mean and SEM of three replicates. **(K)** Nuclear (Nu) and cytoplasmic (Cy) fractions of RWPE1 cells expressing the indicated Flag-tagged proteins were immunoblotted with the indicated antibodies. Histone H3 and tubulin control for proper fractionation. All lanes are from the same exposure of the same blot, but extraneous lanes were removed at the vertical line. **(L)** Chromatin immunoprecipitation (ChIP) of ERG or ERG-S215 at three target loci. Two biological replicates (R1 and R2) are shown. Enrichment is the ratio of the copy number of the target loci to the mean copy number of two negative control loci in the same ChIP sample, as measured by quantitative PCR. All P values in this figure by *T*-test (* < 0.01, ** < 0.005, *** < 0.001).

ERG is not normally expressed in prostate cells. However, in 50% of prostate cancers, a chromosomal rearrangement of the *ERG* gene results in high ERG expression [14]. ERG expression in prostate cells has been shown to increase cellular migration and invasion [15,43]. In a transwell migration assay, ERG over-expression increased RWPE1 cell migration, but a mutation of the ERK phosphorylation site (S215A) completely abolished this function (Figure 4A to C). Similarly, mutation of the DEF domain sequence (FIFP to AAAP) abolished the ability of ERG to drive cell migration (Figure 4D to F). Likewise, ERGi10, which lacks S215 phosphorylation (Figure 3E) could not induce RWPE cell migration (Figure 4G to I).

As a transcription factor, the role of ERG in cell migration is likely due to regulation of target gene expression. To test the role of S215 phosphorylation in ERG transcriptional function, we compared the levels of *PIK3AP1* and *ARHGAP29*, two ERG target genes [15] with known cell migration and oncogenic functions [44,45]. Both genes were activated about three-fold by ERG expression in RWPE1 cells, but neither were activated by an ERG S215A mutant (Figure 4J). Therefore ERG S215 phosphorylation is required for activation of ERG target genes in prostate cells.

The inability of ERG S215 to activate target genes could be due to a defect in subcellular localization, genomic occupancy, or transactivation function. To test localization, nuclear and cytoplasmic fractions of RWPE cells expressing ERG or ERG-S215A were immunoblotted. ERG was found to be in both the cytoplasm and the nucleus, and this distribution was unchanged in ERG-S215A (Figure 4K). Chromatin immunoprecipitation of ERG or ERG-S215A revealed a trend of reduced occupancy for ERG-S215A, compared to ERG, at three target loci (Figure 4L). This finding indicates that S215 phosphorylation may play a role in the interaction of ERG with chromatin.

### A high affinity ERK-ERG interaction allows ERG function when there is low ERK signaling

**Figure 5:**
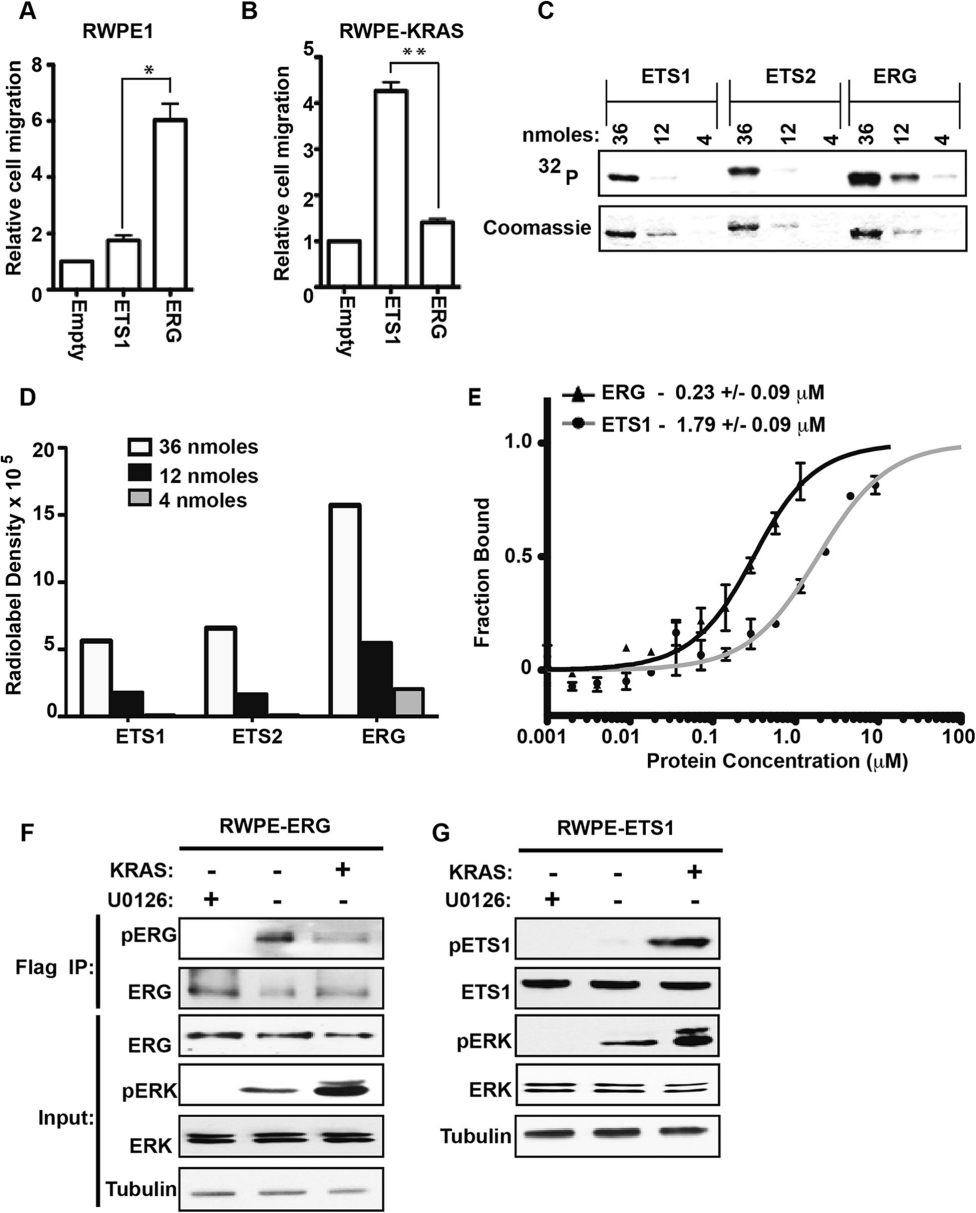
**ERK has higher affinity for ERG than ETS1 and ERG is phosphorylated in cells with lower pERK levels. (A, B)** A transwell assay compared cell migration of RWPE1 and RWPE-KRAS (RWPE2) cell lines over-expressing the indicated ETS protein compared to empty vector. Mean and SEM of three biological replicates are shown. P-values (* < 0.01, ** < 0.005) by *T*-test. **(C)** Indicated amount of purified ETS1, ETS2 and ERG were phosphorylated in vitro by ERK2 kinase. Coomassie staining indicates total protein and an autoradiograph, phosphorylation. **(D)** Quantification of radiolabel density in **(C)** in arbitrary density units. **(E)** The binding of fluorescently labeled ERK2 to varying concentrations of ERG or ETS1 by microscale thermophoresis (MST). **(F)** Immunoblots with indicated antibodies of cell extracts or Flag-immunoprecipitate from cells stably over-expressing ERG in the presence of the MEK inhibitor U0126, or over-expressed KRAS, as indicated. **(G)** Immunoblots as in **F**, but with cells stably over-expressing ETS1.

We have previously shown that both ERG and the ubiquitously expressed ETS protein, ETS1, can bind the ETS/AP-1 RAS-response element in the enhancers of cell migration genes, including *PIK3AP1* and *ARHGAP29*, but have distinct roles in cell migration depending on RAS/ERK signaling status [13,15]. In RWPE1 prostate cells, ERG, and not ETS1, promotes cell migration (Figure 5A). However, the over-expression of KRAS in RWPE1 cells (RWPE-KRAS, also known as RWPE2), allows ETS1 and not ERG to promote cell migration (Figure 5B). Table 1 indicates that ERG is a more robust ERK target than ETS1. If this difference allows ERG to function in cells with low levels of pERK (RWPE1), while ETS1 can only function in cells with high levels of pERK (RWPE-KRAS), it would explain the difference in Figure 5A and B.

To more quantitatively assess ERK selectivity, we used three concentrations of purified protein to compare ERK2 phosphorylation of ERG and ETS1 (Figure 5C). ETS1 is known to have two ERK phosphorylation sites (T38 and S41) [46], while ERG has one (S215), or two (S96), so the radiolabel for pETS1 should be higher, or equal to, that of pERG if the kinase targets both proteins similarly. Despite this, quantification of the blot (Figure 5D) indicates approximately three-fold higher phosphorylation of ERG compared to ETS1. As a control, ETS2 was also tested. Consistent with Table 1, there was no difference in ERK2 phosphorylation of ETS1 and ETS2 (Figure 5D).

This higher selectivity could be due to ERK docking with ERG with higher affinity than ETS1. To test this, the affinity of ERK for ERG and ETS1 was compared using microscale thermophoresis (MST) (Figure 5E). The affinity of ERK for ERG (K_D_ = 0.23 μM) was eight times higher than for ETS1 (K_D_ = 1.79 μM), and this difference was statistically significant (P = 0.023, by *T*-test).

These in vitro data suggest that low levels of pERK could phosphorylate ERG, but higher levels would be required to phosphorylate ETS1. To test if this is true in vivo, levels of pERG and pETS1 were compared in the RWPE system. RWPE cells over-expressing ERG (Figure 5F) or ETS1 (Figure 5G), had low levels of pERK. Treatment with the MEK inhibitor U0126 further reduced pERK levels, and over-expression of KRAS increased pERK levels (Figure 5F and G). Phosphorylation of ETS1 was only observed in cells with high levels of pERK (Figure 5G, RWPE-KRAS), consistent with ETS1 promoting cell migration in only this cell line (Figure 5B). In contrast, ERG was phosphorylated in RWPE1 cells, and ERG phosphorylation did not increase in RWPE-KRAS cells (Figure 5F), indicating that low levels of pERK are sufficient for peak ERG phosphorylation in vivo. There was actually a small decrease in ERG phosphorylation in RWPE-KRAS cells indicating the potential for additional feedback mechanisms controlling this modification. These findings are consistent with a model that due to a high-affinity ERK interaction, ERG functions in prostate cells with low level ERK signaling and ETS1 functions in prostate cells with high ERK signaling. These results indicate that a direct comparison of MAPK function between ETS proteins, as shown in Figure 1, can inform in vivo mechanisms.

## Discussion

The ETS family DNA binding sequence (consensus CCGGAAGT) occurs in multiple cis-regulatory motifs that mediate the gene expression changes that occur after mitogen signaling [47,48]. However, the size of the ETS family has made it difficult to determine all of the ETS factors that could potentially activate or repress these genes. Here we provide the relative specificity of MAPKs across the ETS family of transcription factors. These data allow predictions of changes in signaling response that could occur when one bound ETS factor is replaced by another, either due to changes in expression levels, subcellular localization, or DNA affinity. We provide one example of the type of findings that can result from this dataset: The novel, high-affinity interaction of ERK with ERG that provides a potential explanation for how ERG can promote migration of prostate cells with low levels of RAS/ERK signaling, but ETS1 cannot.

Comparing MAPK targeting of ETS factors provided data consistent with previous studies (Tables 1, 2 and 3), but also puts these studies into perspective within the greater ETS family. The ternary complex factor (TCF) subfamily of ETS factors including ELK1, ELK3 (NET), and ELK4 (SAP1) are among the best-studied MAPK targets [49]. These factors bind to serum-response elements due to a cooperative interaction with SRF and mediate immediate early gene expression [50]. ELK1 is phosphorylated at multiple sites by all three MAPK classes [20,51]. We found that ELK1 was, by far, the most phosphorylated ETS protein by ERK2 and JNK1 in vitro, and is the second most phosphorylated by p38α. ELK4 is known to be a better substrate for ERK and p38 kinases [34], than for JNK [47], and our findings agreed (Figure 1). ELK3 can also be a target for all three MAPKs [24], but we found lower levels of phosphorylation compared to the other two TCFs. ELK3 also had specificity for ERK2, likely due to a DEF domain. Another interesting finding involved ETV3, which has been recently identified as a target of ERK2 [8]. We found that ETV3 was indeed the fifth most phosphorylated ETS by ERK2 (Table 1). But ETV3 was also a very strong JNK1 and p38α substrate, ranking second and first, respectively, within the ETS family, indicating a potential role for these pathways in ETV3 function. A few ETS proteins previously reported as MAPK targets showed very little phosphorylation in our assay (Figure 1). These discrepancies may be due to differences in enzyme concentration. For example, a concentration of ERK2 400-fold higher than used here was reported to phosphorylate GABPA [30]. These differences highlight the utility of comparing all members of the family at the same time, and in the same conditions.

The number of PX(SP/TP) motifs correlated with targeting by all MAPKs, and particular D and DEF domain sequences correlated with specificity for p38 and ERK. However, these sequences alone could not entirely predict our findings. This is likely because of the role of tertiary structure in kinase interactions with both the docking sequence and the target residues. In the case of ETS1 and ETS2, for example, the structure of the pointed domain can mediate a specific ERK2 interaction [52], consistent with the ERK2 specificity we observed in Figure 1.

Our survey discovered ERK phosphorylation of S215 in ERG, and a specific antibody confirmed that this phosphorylation event occurs in vivo (Figure 3). We demonstrated that this phosphorylation event was required for ERG to promote prostate cell migration. ERG is normally expressed in hematopoietic and endothelial cells [4], but is aberrantly expressed in about one-half of prostate cancers due to a chromosomal rearrangement [14]. An early study indicated that ERG is heavily phosphorylated in myeloid cells, but these sites have not been characterized [53]. S215 phosphorylation has been observed by mass spectrometry of ERG immunoprecipitated from VCAP prostate cancer cells [42], indicating that this residue is phosphorylated in cells with the TMPRSS2:ERG fusion. However, neither the kinase responsible, nor the importance of the phosphorylation event was previously characterized. Our findings indicate that ERK phosphorylates ERG through an interaction with the DEF domain and this modification is critical for ERG to promote prostate cell migration. This phosphorylation site was found to be important for activation of ERG target genes (Figure 4J) and for ERG chromatin occupancy (Figure 4L). However, whether this effect is due to alteration of a protein-DNA or protein-protein contact is unknown and will need to be determined in future studies.

The higher affinity of ERK2 for ERG compared to ETS1 correlated with the finding that ERG S215 is phosphorylated in cells with low levels of ERK signaling, while ETS1 T38 is only phosphorylated in cells with high levels of ERK signaling. It also corresponds to the finding that ERG can promote cell migration in a low ERK signaling background, but ETS1 requires a high ERK signaling background (Figure 5). These data led us to hypothesize that the difference in affinity of ERK for ERG versus ETS1 mediates the difference in biological function. However, there are other possible explanations. For example, p38 and JNK can be activated by KRAS signaling and these kinases are more specific for ETS1 than ERG (Figure 1). Therefore, these kinases could play a role in ETS1 function when KRAS is activated. Also, kinases downstream of ERK [54], such as RSK1 and MSK1, would be sensitive to MEK inhibition and could play a role in ERG or ETS1 function.

The ability of ERG to drive cell migration in a background of low, but not high RAS/ERK signaling provides an interesting example of an oncogene that actually requires low levels of an oncogenic signaling pathway. We found that ERG can promote an oncogenic phenotype, cell migration, in RWPE1 cells. RWPE1 cells are grown in media containing recombinant epidermal growth factor (EGF), which activates RAS/ERK signaling, but because the RAS/ERK pathway is intact, negative feedback loops can keep ERK activity low [55]. In a tumor, similar, low level ERK activity could occur through autocrine signaling, or signaling from the microenvironment. Consistent with this model, increases in growth factor production are common in prostate cancer [56], but mutations that disrupt RAS/ERK feedback and lead to very high ERK activity are rare [57]. Further supporting this model, we have shown that these rare activating mutations in RAS or RAF are mutually exclusive with *ERG* gene rearrangements in prostate tumors [16]. As the *TMPRSS2-ERG* fusion is the most common genomic alteration in prostate cancer, and cell migration is a key component of metastasis, the high-affinity ERK2/ERG interaction represents a potential target for small molecule inhibition during prostate cancer treatment.

## Conclusions

Here we characterize the MAPK specificity across the ETS family of transcription factors. Phosphorylation by all three MAPKs tested correlated with the number of PX(TP/SP) motifs, while p38α specificity correlated with LRL motifs and ERK2 specificity with FXFP motifs. A novel ERK2 phosphorylation site was identified in ERG. Phosphorylation of this site via a high affinity interaction with ERK2 was necessary for ERG function in prostate cells and indicates that low level RAS/ERK signaling may be required for ERG to drive prostate cancer.

## Materials and methods

### Cloning and protein purification

SPIB, ETV7, ELF3 (25728) and ELK1 (27156) clones were obtained from Addgene or were gifts. ETV2 mRNA-ORF clone was from GeneCopoeia (EX-H1877-M02), and ETV3L from Origene (RC22031). All other ETS clones were reverse transcribed (RT-PCR) from mRNA isolated from cell lines or tissues with high expression [4]. ETS open reading frames were cloned into pET28a (Novagen) using oligonucleotides in (Additional file 1: Table S1) and sequence verified. 6X His-tagged proteins were induced in BL21 pRIL with 1 mM IPTG at 37°C for 2 h. Cells were pelleted and lysed by sonication in extraction buffer (50 mM Tris, pH 7.9, 1 mM EDTA, 1 M KCl, 1 mM DTT), and centrifuged. Inclusion bodies were washed with extraction buffer, and solubilized in urea-lysis buffer (10 mM Tris, pH 7.9, 750 mM NaCl, 5 M urea, 0.1% Triton-x-100, 20 mM imidazole) by sonication. Soluble fraction was incubated with Ni-NTA agarose resin (Qiagen) and rotated overnight at 4°C, washed 6 times with urea-lysis buffer and eluted with urea-lysis buffer with 750 mM imidazole. Protein was refolded by dialysis into TGEK200 (10 mM Tris, pH7.9, 0.5 mM EDTA, 200 mM KCl, 1 mM DTT and 10% glycerol). Concentration was calculated by comparison to BSA standards on coomassie SDS-PAGE gels. DNA binding activity was verified by EMSA using the sequence: 5′ GCCACGGCCCAGGAAGTGACTCACCCACCCTGATG as previously described [5]. ERK2 enzyme was co-expressed with constitutively active MEK to create pERK2, and purified from bacteria as described [52]. JNK1 and P38α were from SignalChem.

### Phosphorylation of ETS proteins

Kinase reactions were performed as described previously [10] with some modifications. In brief, kinase reactions were 30 min at 30°C in 20 μl of buffer containing 5 nM kinase, 1.5 μM ETS protein, 25 mM Tris pH7.9, 1 mM Dithiothreitol (DTT), 10 mM Magnesium Acetate, 2 mM ATP, 12 mM β- glycerophosphate, 0.5 mM Na3VO4, 5% glycerol, 87.5 mM KCl, and 8 μCi of 3 Ci/μmole γ-^32^P ATP. The kinase assays shown in the left panels of Figure 2C also included 0.5 mg/ml bovine serum albumin (BSA). Reactions were stopped by the addition of 4% SDS and electrophoresed on a 12.5% SDS-Poly acrylamide gel. Gels were stained with coomassie blue and radioactivity was detected by PhosphorImaging (Amersham Biosciences Typhoon 9210) and quantified using ImageQuant TL. Density of coomassie stain was measured using ImageJ 1.48v.

### Cell culture and transwell migration assays

RWPE1 and RWPE2 cell lines were cultured in Keratinocyte SFM (Invitrogen) and 1% Penicillin/Streptomycin (100X solution – Mediatech-Cellgro). ETS proteins with N-terminal 3xFlag tags were stably expressed in RWPE1 or RWPE2 cells via retrovirus as described previously [15]. Transwell migration assays were done as described previously [15]. In brief, 5×10^4^ cells were introduced to the transwell (8 μM pore size; BD Bioscience) and incubated for 60 h. Migrated cells are reported as the mean of at least three biological replicates with two technical replicates each.

### Measuring RNA, protein immunoblotting, and ChIP

RNA levels were measured by quantitative RT-PCR as described previously [15] using primers in (Additional file 1: Table S1). Total Protein extract from equal number of cells were separated on 10% SDS-PAGE gels and immunoblotted by standard procedures. ERK (sc-94), Phospho ERK (sc-7383), ETS1 (sc-350) antibodies were from Santa Cruz Biotechnology. Tubulin (T9026) and Flag (F1894) antibodies were from Sigma. Phospho- (T38) ETS1 (ab59179) was from Abcam. ERG (9FY) antibody was from Biocare Medical. Phospho-specific antibody for ERG S215 (pERG) was produced by Pierce Custom Services, using the phosphorylated peptide DKALQN(pS)PRLMHAR. ChIP from RWPE1 cells was performed as previously described [15] using an ERG antibody (Biocare Medical, CM421A). ChIP enrichment was assessed using quantitative PCR and standard curves to measure copy number of the locus of interest and two negative control loci. Primer sequences are included in (Additional file 1: Table S1).

### Inhibitor treatments and immunoprecipitation

Prior to immunoprecipitation, cells were treated for 6 hours with the following inhibitors: MEK1/2 (10 μM, U0126, Cell Signaling), JNK (50 μM, SP600125, Cell Signaling), P38 kinase (10 μM, SB203580, Cell Signaling), AKT (20 μM, AKT inhibitor VIII, Santa Cruz Biotechnology), PKA (40 μM, H-89 Dihydrochloride, Cell Signaling), PI3Kinase (20 μM, LY294002, Cell Signaling), FRAP/mTOR (40 nM, Rapamycin), or DMSO,and cells were washed twice with ice cold 1X PBS and solubilized in RIPA buffer (50 mM Tris–HCl, pH7.4, 150 mM NaCl, 1 mM EDTA, 1% NP-40, 1% Sodium Deoxycholic acid, 0.1% SDS, 50 mM β- glycerophosphate, 10 mM NaF, 0.5 mM DTT). Equal concentration of protein extracts were added to the anti Flag M2 magnetic beads (Sigma) and rotated overnight. Beads were washed 3 times with 1 M RIPA buffer and immunoblotted.

### Microscale thermophoresis (MST)

MST analysis used a NanoTemper Monolith NT.115 (NanoTemper Technologies) as described [58]. Briefly, purified ERK was fluorescently labeled using an amine reactive L001 Monolith Protein Labeling Kit RED-NHS (NanoTemper Technologies). 200 nM ERK2 was incubated for 30 minutes at room temperature in the dark with different concentrations of purified ETS1 or ERG protein. The samples were loaded into standard glass capillaries (Monolith NT capillaries, NanoTemper Technologies) and thermophoresis analysis was performed (LED 80%, IR laser 80%). Dissociation constants were calculated using NanoTemper 1.2.206 analysis program.

## Additional files

Additional file 1: Table S1. Cloned sequences and primers used. Additional file 2: Figure S1. The DNA binding activity of the purified His-tagged ETS proteins. Additional file 3: Figure S2. Coomassie staining of in vitro kinase assay gels. Additional file 4: Table S2. D and DEF domains in ETS proteins.
