# Supplementary material for: Comparison of MAPK specificity across the ETS transcription factor family identifies a high-affinity ERK interaction required for ERG function in prostate cells
Source: Cell Commun Signal. 2015 Feb 19;13:12. doi: 10.1186/s12964-015-0089-7 (PMC4338625; doi:10.1186/s12964-015-0089-7)
Supplement: Additional file 1: Table S1. — Cloned sequences and primers used. [file 12964_2015_89_MOESM1_ESM.pdf]

ETS	Ref seq or GenBank	Isoform	Forward primer	Reverse primer
ETS1	NM_001143820.1	1	ctagaattccatatgaaggcggccgtcgatctcaagc	ataagaatgcggccgctcatcactcgtcggcatctggcttg
ETS2	NM_005239.4	1	ctagaattccatatgaatgatttcggaatcaagaatatgg	tgcggatcctcatcagtcctccgtgtcgggctgg
ERG	NM_182918.3	1	gatccaagtgcgcacgctagcatggccagcactattaaggaagcc	gataagatagcggccgcttagtagtaagtgcccagatgag
ERGv10	KP_100545	10	Same as above	Same as above
FLI1	NM_002017.3	1	agcttccacccaggacatatggacgggactattaaggaggc	gcttaactacgccggcgctagtagtagctgcctaagagtg
FEV	NM_0175211.2		ctagaattccatatgagacagagcggcgcctcccag	tctgcaatgaattcactagtggtaatggccccccaagtgc
ETV3	NM_001145312.1	1	ctagaattccatatgaaagccggctgtagcatcgtggaaaagcc	tctgcaatgaattcactaagcatcagcagctgctgttgccaagcc
ERF	NM_006494.2	1	ctagaattccatatgaagaccccggcggacac	agtacgaagctttcaggagtctcggtgctcc
ELF1	NM_172373.3	a	ctagaattccatatggctgctgttgtccaacagaacg	gtgacattgcggccgctactaaaaagagttgggttccagcagttcg
ELF2	NM_201999.1	1	ctagaattcgctagcatggcgacgtctctgcatgagg	gtgacattgcggccgctattatttctcacatgtcactagtcc
ELF4	NM_001422.2		ctagaattccatatggctattaccctacagcccagtg	gtgacattgcggccgctattatatgtcatggggctccatcttaatgag
ELF3	NM_001114309		ctagaattccatatggctgcaacctgtgagattagc	tctgcaatgaattctcagttccgactctggagaacc
ELF5	NM_001422.2	2	cagtacgctagcatgttggactcggtgacacacag	tctgacggatcctcatagcttgtcttcctgccacc
EHF	NM_012153.3	2	tctgacggatcctcatagcttgtcttcctgccacc	tctgcataggatcctatcagttttcattttctctccatcctc
GABPA	NM_002040.3		ctagaattccatatgactaaaagagaagcagaggagc	ataagaatgcggccgctcatcaattatccttttccgtttgcagagaagc
ELK1	NM_001114123.1	a	ctagaattccatatggacccatctgtgacgctg	tctgacggatcctcatggcttctggggccctg
ELK3	NM_005230.2		ctagaattccatatggagagtgcaatcacgctgtg	agtacgaagctttcaggatttctgagagtttgaagaaag
ELK4	NM_021795.2	b	ctagaattccatatggacagtgctatcaccctgtggcagttc	tctgcaatgaattcttatgtcttctgtaggtctggggaaaatgg
ETV1	NM_004956.4	a	actgtcagtggagctagcatggatggattttatgaccagcaagtgc	atccgccggcgttaatacacgtagccttc
ETV4	NM_001986.2	1	agatcactagtacatatggagcggaggatgaaagccgga	cgagaaacacgcggcgcctagtaagagtagccacccttcc
ETV5	NM_004454.2		ctagcatatggtgctagcatggacgggttttatgatcagcaagtcc	ctatatgcaatccgccggcgttagtaagcaaagccttcggc
ETV2	NM_014209.2	1	ctagaattccatatggacctgtggaactgggatgag	tctgcaatgaattcattattgtgtctctgctccc
SPIB	NM_003121.3	1	atgactccatgggcagcagccatcatcatcatcatcacatgctcgccctggaggctg	tctgcaatgaattctcaggcccggcggactgc
SPIC	NM_152323.1		cagtacgctagcatgacgtctgttgaacaagacaagc	tctgacggatccttagcaatcatggtgatttagctc

SPI1	NM_001080547.1	1	cagtacgctagcatgttacaggcgtgcaaaatggaag	tctagcggatcctcagtggggcgggtggcg
ETV6	NM_001987.4		ctagaattccatatgtctgagactcctgctcagtg	tctgcaatgaattctcagcattcatcttcttggtatatttg
ETV7	NM_016135.2	1	ctagaattccatatgcaggagggagaattggctatttc	tctgcaatgaattctcacggagagatttctggcctc
SPDEF	NM_012391.1	1	gtttgcaagctacatatgggcagcgccagcccgggtctga	ctgaaggtccgccggcgtcagatggggtgcacgaactgg

qRT-PCR Gene	Forward Primer	Reverse Primer
ARHGAP29	ggaatcagaacgcaagcaaaatgcg	gggatgctgattcagcctcttgg
PIK3AP1	tgtgacgatgagccagagac	tcaggttcccaggtgaagtc

ChIP locus	Forward Primer	Reverse Primer
Negative Control 1	ctgccacttgagggtgagg	ccatcttgcatgcagttagcc
Negative control 2	atggttgccactggggatct	tgccaaagcctaggggaaga
SKI	ggagccagtgacctcatttc	gcacagtgtgttgcatacagg
SLC6A13	gtggctgtttgtctccttgc	aagaagagcttgtgcccctc
STK11IP	ctcttccgcttcctctttcc	gagcttccacaacagggagt

Table S1. Primer sequences used in this study. Primers used to clone the indicated ETS protein isoform into pet28a are shown at the top. Sequenced clones matched the indicated Refseq or GenBank record. Primer sequences used for gene expression profiling, and analysis of chromatin immunoprecipitation (ChIP) enrichment are shown below.
