# Supplementary material for: Comparison of MAPK specificity across the ETS transcription factor family identifies a high-affinity ERK interaction required for ERG function in prostate cells
Source: Cell Commun Signal. 2015 Feb 19;13:12. doi: 10.1186/s12964-015-0089-7 (PMC4338625; doi:10.1186/s12964-015-0089-7)
Supplement: Additional file 2: Figure S1. — The DNA binding activity of the purified His-tagged ETS proteins. [file 12964_2015_89_MOESM2_ESM.pdf]

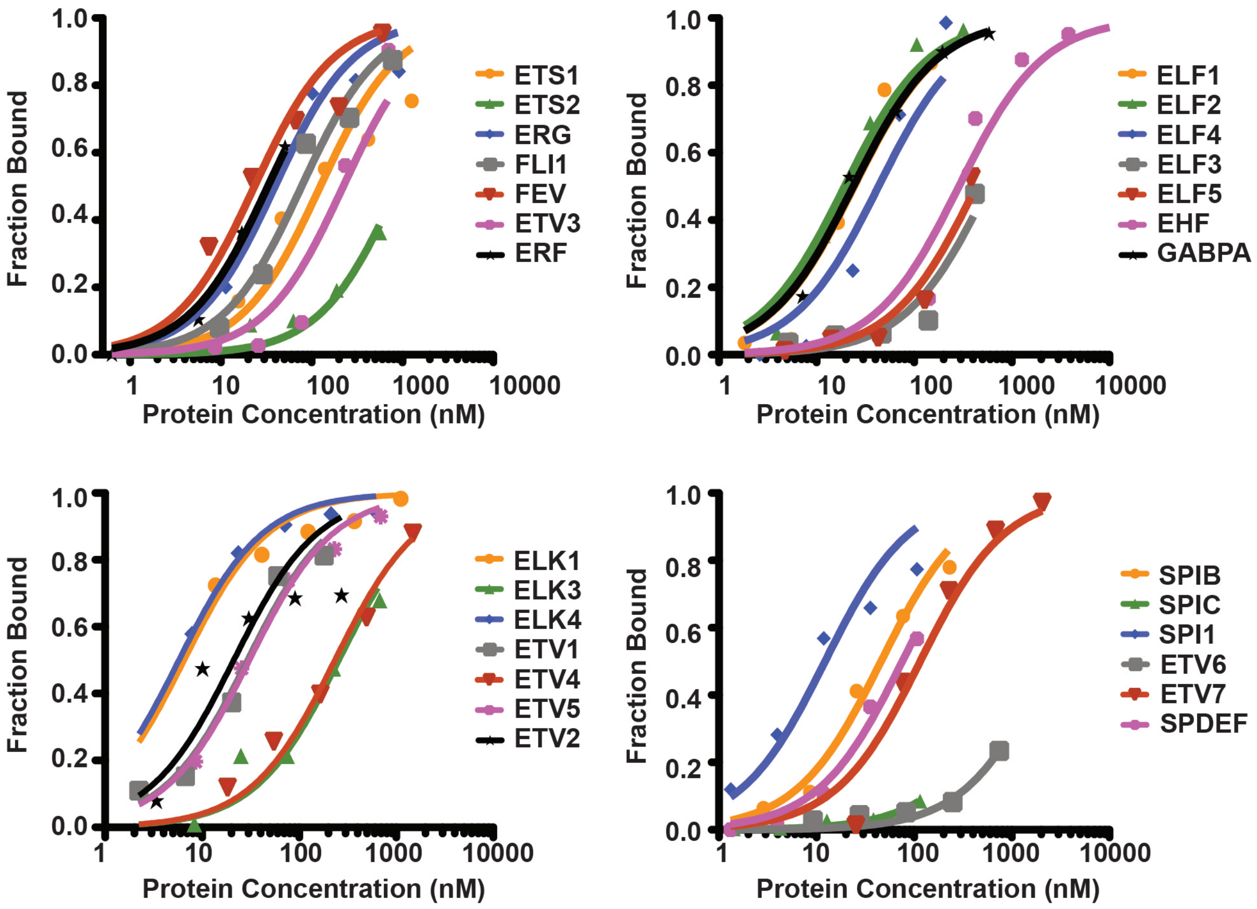

Figure S1. The DNA binding activity of the purified His-tagged ETS proteins. Quantification of an electrophoretic mobility shift assay (EMSA) using the indicated ETS proteins. A radiolabled probe with an ETS consensus binding sequence was incubated with the indicated concentrations of protein. Fraction bound is one minus the ratio of free probe in the indicated lane to free probe in the zero protein lane. Two ETS proteins (SPIC and ETV6) have low affinity, likely due to autoinhibition.
