# Supplementary material for: Comparison of MAPK specificity across the ETS transcription factor family identifies a high-affinity ERK interaction required for ERG function in prostate cells
Source: Cell Commun Signal. 2015 Feb 19;13:12. doi: 10.1186/s12964-015-0089-7 (PMC4338625; doi:10.1186/s12964-015-0089-7)
Supplement: Additional file 3: Figure S2. — Coomassie staining of in vitro kinase assay gels. [file 12964_2015_89_MOESM3_ESM.pdf]

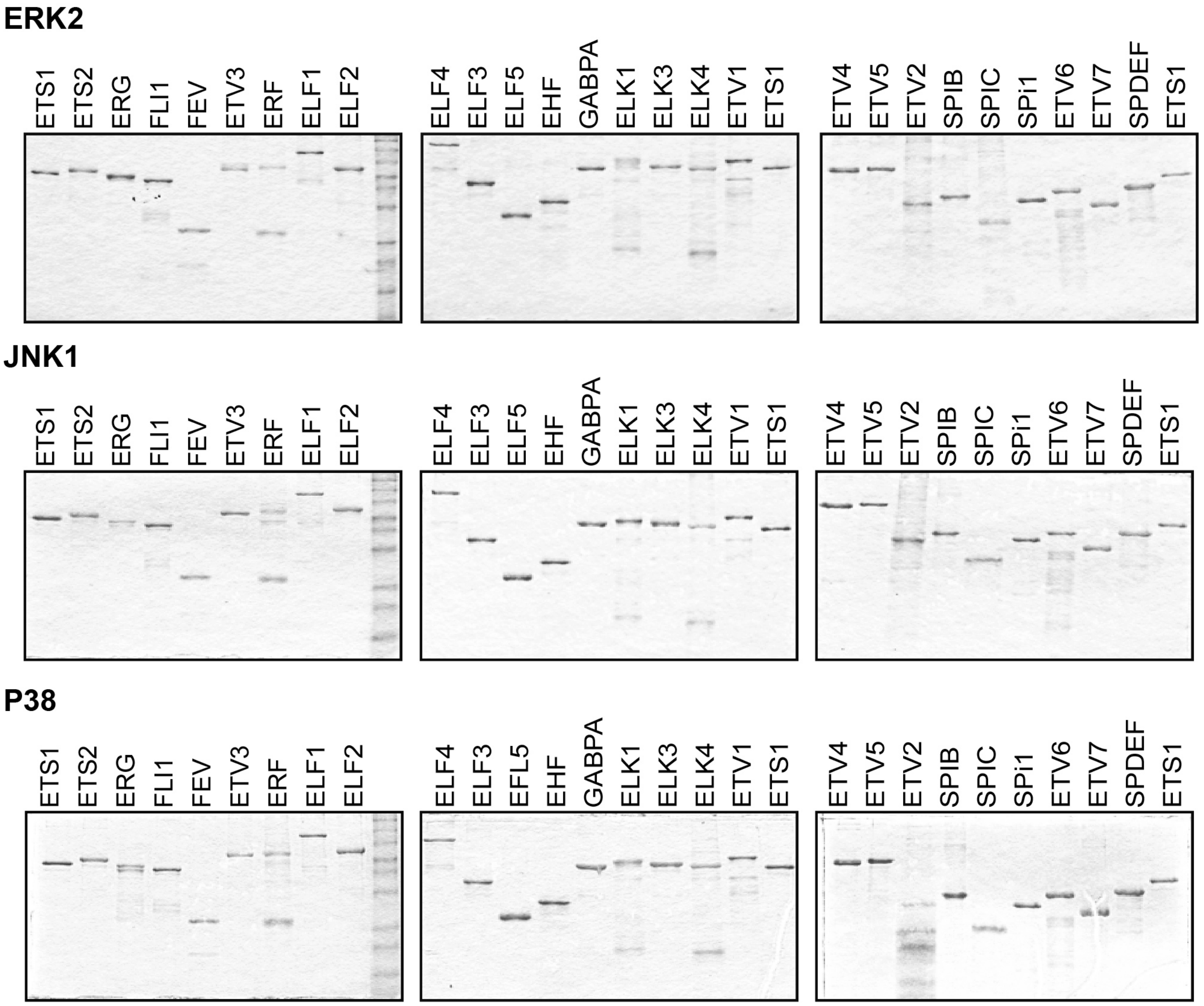

Figure S2. Coomassie staining of in vitro kinase assay gels. The same gels used for each autoradiograph in Figure 1 are stained with coomassie and shown here. The band corresponding to the full-length protein was used for quantification of total protein.
