# Supplementary material for: Comparison of MAPK specificity across the ETS transcription factor family identifies a high-affinity ERK interaction required for ERG function in prostate cells
Source: Cell Commun Signal. 2015 Feb 19;13:12. doi: 10.1186/s12964-015-0089-7 (PMC4338625; doi:10.1186/s12964-015-0089-7)
Supplement: Additional file 4: Table S2. — D and DEF domains in ETS proteins. [file 12964_2015_89_MOESM4_ESM.pdf]

ETS	D domain seq	LXL seq	DEF domain seq
ETS1	PTLTIIKTEKVDLELFPSP	LTI	
	MNGAALCALGKDCFLELAP	LEL	
	GYTPEELHAMLDVKPDADE	LDV	
ETS2	GQMLCNLGKERFLELAPDF	LEL	
	PKSRLSSVSVTYCSVSQDF	VSV	
ERG	PAAHIKEALSVVSEDQSLF	LSV	FIFP
	PHPPALPVTSSSFFAAPNP	LPV	
FLI1	PHPSSMPVTSSSFFGAASQ	MPV	
	MDGTIKEALSVVSDDQSLF	LSV	
FEV	SWGPLSPAVQKGSGQIQLW	IQL	FDF
ETV3	PIPGRGGVLNVPISPALSL	LNV	FHFP
	GIGHQKRKPDIMLPLFARP	IML	FNF
	SSPGSRQIQLWHFILELLQ	IQL	FSF
	YHLSPRTFPRYPGLMVPPL	LMV	
ERF	VSSDLQHATAQLSLEHRDS	LSV	FSF
	SSPGSRQIQLWHFILELLR	IQL	FRFP
			FAFP
			FKF
			FNF
ELF1	NSKAAKPKDPVEVAQPSEV	VEV	FEF
	DDMVVAPVTHVSVTLDGIP	VSV	
	KKNKDGKGNTIYLWEFLLA	IYL	
	HTVTLQTVPLTTVIASTDP	VPL	
	IPSSQPMTVLKENVMLQSQ	MTV	
	KENVMLQSQKAGSPPSIVL	VML	
ELF2	SVSATAAPRTVRVAMQVPV	VRV	
	PTCLRDSRSPVEVFVPPCV	VFV	
	PLAVRALTPVSIAHGTPVM	LAV	
	AVAKKQEHDVKTLQLVEEK	LQL	
ELF4	QTLSPPSRPTVGLTPVAEL	VGL	
	KIQHVGLQPSASLELGPSL	VGL	
	EQVPYPDLLHLYSGLELDD	LHL	
	KKSKDGKGSTIYLWEFLLA	IYL	
ELF3			FKF
ELF5			
EHF	CRAQISMTTTSHLPVAESP	ISM	FRF
	NGEHLCSMSLQEFTRAAGT	MSL	
GABPA	ILEIVKPADTVEVVIDPDA	LEI	
	DQGVKTDGTVQLSVQVISY	VQL	
ELK1	QPQKGRKPRDLELPLSPSL	LEL	FQFP
	LNVEPGLGRALPPEVKVEG	VKV	
ELK3	SVSAKISSLMLPNAASISS	LML	FQFP
	PTLLNGHMPVPIPSLDRAA	MPV	
	PPKAKKPKGLEISAPPLVL	LEI	
	PEILKMDPHAVEISRESLL	LKM	
ELK4	NSSRSKKPKGLELAPTLVI	LEL	FQFP
ETV1	YDQKPQVGMRPSNPPTPSS	VGM	FKF
	PTYQRRGSLQLWQFLVALL	LQL	
	YQAESLAFHGLPLKIKKEP	LPL	
ETV4	PPYQRRGALQLWQFLVALL	LQL	
	RQIAIKSPAPGALGQSPLQ	IAI	
ETV5	PPYQRRGSLQLWQFLVTLL	LQL	
	PHQPLQMPKMMPENQYPSE	LQM	
	GPPAHGFQSPMGIKQEPRD	MGI	
ETV2	TSLKRYQSSALTVCSEPSP	LTV	FCFP
	ASLARCPKTNHRGPIQLWQ	IQL	
SPIB			FQF

SPIC	STGDLQYSPDYRNYLALIN	LAL	FQF
SPI1	EGERQSPPLEVSDGEADGL	LAL	FQF
ETV6	PVPRALRMEEDSIRLPAHL	LRM	FRF
	GLHREGKPINLSHREDLAY	INL	
	VSVSPPEEHAMPIGRIADC	MPI	
ETV7	ARWTPGKEESLNLCHCAEL	LNL	FRF
	THVQARCEAQINLLGEGGI	INL	
SPDEF	DTVSRTGLEKAAAGAVGLE	VGL	
	RSPLGGDVLHAHLDIWKSA	LDI	

Table S2. D and DEF domains in the ETS proteins. D domains were identified using the D-finder algorithm with no cutoff value. The only D domains with a Log(P^v) of less than 23 are the first listed domains for ELK1, ELK3, ELK4, and ETS1. DEF domains were identified by sequence scanning for FXF or FXFP motifs.
